# Moderate-Intensity Exercise Enhances Mitochondrial Biogenesis Markers in the Skeletal Muscle of a Mouse Model Affected by Diet-Induced Obesity

**DOI:** 10.3390/nu16121836

**Published:** 2024-06-12

**Authors:** Lauren Jun, Emily Knight, Tom L. Broderick, Layla Al-Nakkash, Brielle Tobin, Thangiah Geetha, Jeganathan Ramesh Babu

**Affiliations:** 1 Department of Nutritional Sciences, Auburn University, Auburn, AL 36849, USA; lzj0047@auburn.edu (L.J.);; 2 Department of Physiology, College of Graduate Studies, Midwestern University, Glendale, AZ 85308, USA; 3 The Arizona College of Osteopathic Medicine, Midwestern University, Glendale, AZ 85308, USA; 4 Boshell Metabolic Diseases and Diabetes Program, Auburn University, Auburn, AL 36849, USA

**Keywords:** obesity, skeletal muscle, mitochondria, exercise, high-fat diet

## Abstract

Skeletal muscle is composed of bundles of muscle fibers with distinctive characteristics. Oxidative muscle fiber types contain higher mitochondrial content, relying primarily on oxidative phosphorylation for ATP generation. Notably, as a result of obesity, or following prolonged exposure to a high-fat diet, skeletal muscle undergoes a shift in fiber type toward a glycolytic type. Mitochondria are highly dynamic organelles, constantly undergoing mitochondrial biogenesis and dynamic processes. Our study aims to explore the impact of obesity on skeletal muscle mitochondrial biogenesis and dynamics and also ascertain whether the skeletal muscle fiber type shift occurs from the aberrant mitochondrial machinery. Furthermore, we investigated the impact of exercise in preserving the oxidative muscle fiber types despite obesity. Mice were subjected to a normal standard chow and water or high-fat diet with sugar water (HFS) with or without exercise training. After 12 weeks of treatment, the HFS diet resulted in a noteworthy reduction in the markers of mitochondrial content, which was recovered by exercise training. Furthermore, higher mitochondrial biogenesis markers were observed in the exercised group with a subsequent increase in the mitochondrial fission marker. In conclusion, these findings imply a beneficial impact of moderate-intensity exercise on the preservation of oxidative capacity in the muscle of obese mouse models.

## 1. Introduction

Skeletal muscle is the largest organ in the body, comprising up to 50% of total body mass in a healthy-weight individual [1]. This organ is heterogenous in nature and composed of bundles of muscle fiber with unique characteristics based on their metabolic and contractile properties [2]. The variation of these specific muscle fiber types is mainly based on myosin heavy chain (MyHC) isoforms [2]. In mammals, the spectrum of muscle fibers includes three major muscle fiber types, including slow-twitch oxidative fibers (type I), fast-twitch intermediate fibers (type IIA), and fast-twitch glycolytic fibers (type IIB/X). Generally, oxidative fibers have the highest capacity for oxidative metabolism due to higher mitochondrial content, allowing low fatigability [1,3]. In contrast, glycolytic fibers have a preference for glucose as a substrate through anaerobic glycolysis, have a higher fatigability, and exhibit the lowest mitochondrial content [1,3]. In the context of physical activity, oxidative muscle fibers excel during low-impact endurance exercises. Conversely, glycolytic fibers are more engaged during activities that require short bursts of intense energy, such as weightlifting and resistance training [3]. 

Not only is skeletal muscle responsible for physical locomotion, but it is also considered to be one of the major nutrient metabolism sites in the body. Hence, obesity-induced metabolic dysfunction in skeletal muscle can promote metabolic inflexibility, ectopic lipid accumulation and accumulation of toxic lipid intermediates [4], and reduced type I fibers [5]. For instance, obesity is associated with dysregulated glucose uptake and lipid metabolism in skeletal muscle, through mechanisms involving, in part, mitochondrial dysfunction [6]. Specifically, many researchers have demonstrated that obesity leads to reduced mitochondrial content and oxidative capacity with concomitant increases in oxidative stress and intramuscular triglyceride levels in skeletal muscle [6,7]. 

Mitochondria are highly dynamic organelles which undergo constant changes and regulation to maintain cellular homeostasis. These constant regulations of their morphology and structure allow for maintenance of a healthy intracellular mitochondrial pool, where any dysfunctional organelle is targeted for specialized autophagic degradation, known as mitophagy. Additionally, having their own genomic DNA (mtDNA), mitochondria can grow in size and replicate, a process known as mitochondrial biogenesis. The tight regulation of these two contrasting processes is important for cellular adaptation, responding to cellular metabolic state, stress, and other internal and external signals [8]. Hence, an imbalance between these processes is implicated in the onset of numerous pathological conditions [8]. Notably, obesity has been associated with alterations in these mitochondrial systems. For instance, reduced activity of citrate synthase, an important enzyme in the mitochondrial energy system, has been reported in subjects affected by obesity and insulin resistance [9]. Considering the impact of mitochondrial content in oxidative muscle fibers, alterations of the mitochondrial systems have been identified as a factor contributing to the loss of type I fibers in obese individuals [10,11,12]. However, whether the alteration in muscle fiber composition is a cause or consequence of obesity remains elusive. 

Moderate-intensity exercise, which involves low-impact physical activities, induces mitochondrial biogenesis and results in a fast-to-slow fiber type switch [13]. On the other hand, disuse and denervation cause reduced oxidative metabolism and promote slow-to-fast fiber type switch [13]. Through extensive studies on the identification of several transcription factors and coactivators involved in exercise-induced mitochondrial biogenesis, several key triggering signals have been recognized. The most well-studied signaling molecules include AMP-activated protein kinase (AMPK) [14], peroxisome proliferator-activated receptor (PPAR)-γ coactivator-1α (PGC-1α), nuclear respiratory factor-1 (NRF-1), NRF-2, mitochondrial transcription factor (TFAM), and PPARs [15]. However, this process has traditionally been explored quantitatively, and signaling pathways responsible for the development of fiber-type-specific mitochondrial phenotypes remain poorly understood [15]. 

In this study, we examined the impact of diet-induced obesity in altering muscle fiber type composition. Given the inconsistent effects of moderate-intensity exercise training on muscle fiber phenotype in humans affected by obesity [11], we also examined the effect of moderate-intensity exercise in potentially protecting the slow-twitch fiber profile in the gastrocnemius (GAS) muscle of an obese phenotype of C57BL/6 mouse strain, known for its sensitivity to diet-induced obesity [16]. Obese individuals are reported to be less physically active than normal-weight counterparts [17]. Given that moderate-intensity exercise can impact mitochondrial health and number in skeletal muscle, our study hypothesized that moderate-intensity exercise can prevent the loss of mitochondrial number and function in a model of diet-induced obesity. 

## 2. Materials and Methods

### 2.1. Animals

Four-week-old male C57BL/6 mice were purchased from Charles River (Wilmington, MA, USA). A total of 23 mice were included in this study. Female mice were not involved in this study as they have been shown to exhibit higher type I fibers and endurance exercise capacity compared to male mice [18]. After one week of acclimation, mice were randomly divided into four groups with five-to-six mice in each group: Ln + Sed, Ln + Ex, HFS + Sed, and HFS + Ex. Mice were initially housed in groups of three per cage and kept at 22 °C with 12:12 h light–dark cycle. Body weight was measured weekly and at the beginning and end of the study period. At the time of tissue harvest, mice were asphyxiated in an atmosphere of 100% CO_2_, followed by surgical thoracotomy inducing pneumothorax. Gastrocnemius (GAS) muscles were immediately extracted, frozen in liquid nitrogen, then stored at −80 °C for Western blot and citrate synthase activity assay or fixed in 4% paraformaldehyde for paraffin-embedded tissue staining. The Midwestern University Institutional Animal Care Use Committee approved all experimental procedures (protocol # 3019). Animal care was conducted according to the National Institutes of Health’s Guide for the Care and Use of Laboratory Animals.

### 2.2. Diet Protocol

All mice were given food and water ad libitum. Mice fed a standard rodent diet (Teklad 2918, Inotiv) and tap water served as controls (Ln), while mice fed a high-fat diet with sugar-sweetened water served as obese models (HFS). The high-fat diet contained 20% carbohydrates, 20% protein, and 60% of total calories from fat, with 32.3 g/kg of corn oil (polyunsaturated fat) and 316.6 g/kg lard (saturated fat) (#104239, Dyets Inc., Bethlehem, PA, USA). The sugar-sweetened tap water (42 g/L) consisted of 55% fructose and 45% sucrose. An overnight fast was implemented prior to tissue harvest. 

### 2.3. Exercise Training Protocol

Exercise training involved daily treadmill running (Exer 3/6, Columbus Instruments, Columbus, OH, USA) between zeitgeber (ZT) 0 and ZT3, for 30 min at 12 m/min, five days per week, for a total duration of 150 min per week, as previously described [19]. Briefly, mice were acclimated to daily 10 min sessions of treadmill running for the first week. After the acclimation period, week 1 consisted of 10 m/min for 15 min, and week 2 consisted of 30 min at 10 m/min. From week 3 until week 12, the intensity of exercise was increased to 12 m/min for a 30 min duration, corresponding to an estimated oxygen consumption of ~45–48 mL/kg/min according to the treadmill belt speed [19]. This exercise protocol was designed to mirror the physical activity guidelines stated by the American Diabetes Association, with the recommendation of at least 150 min/week of physical activity [20]. Mice in the running group rested for a period of 48 h before tissue harvest.

### 2.4. Western Blot

Every 10 mg of the frozen GAS sample was lysed with 100 μL of 1× RIPA buffer (#89900, Thermo Fisher Scientific, Rockford, IL, USA) with the addition of a protease inhibitor cocktail (#P8340, Sigma, Kawasaki, Japan) and a phosphatase inhibitor cocktail (#78420, Thermo Fisher Scientific, Rockford, IL, USA) in 1:100 dilution. The lysed samples were centrifuged at 12,000 rpm for 20 min at 4 °C. The supernatant lysates were collected, and protein concentration was analyzed using Pierce™ protein assay reagent (#22660, Thermo Fisher Scientific, Rockford, IL, USA). The lysate samples were heated in 2X Laemmli buffer. A total of 15 μg of total protein was loaded and separated in 10% and 13% polyacrylamide gels and then transferred onto a 0.45 μm polyvinylidene difluoride (PVDF) membrane (#88518, Thermo Fisher Scientific, Rockford, IL, USA). The membranes were blocked for one hour in 5% non-fat milk in tris buffered saline with Tween 20^®^ (TBS-T; 20 mM Tris, 150 mM NaCl, 0.1% Tween20^®^) at room temperature and then incubated with an appropriate primary antibody (Supplementary Table S1) at 4 °C overnight. After three washes in TBST, immunoblots were incubated with horseradish peroxidase (HRP)-conjugated anti-mouse IgG (#ADI-SAB-100-J, Enzo Life Sciences, Farmingdale, NY, USA) or anti-rabbit IgG (#ADI-SAB-300-J) in 1:3000 to 1:7000 dilution at room temperature followed by three washes in TBST and a chemiluminescence detection. Equal loading of protein was verified with GAPDH. The protein expression from two independent experiments was quantified using ImageJ software, v1.54i (National Institute of Health, USA). Densitometry analyses represent the average of three biological replicates. 

### 2.5. Citrate Synthase Activity Assay

Citrate synthase activity in GAS homogenate was measured using MitoCheck Citrate Synthase Activity Assay Kit (#701040, Cayman Chemicals, Ann Arbor, MI, USA), according to the manufacturer’s instructions. Briefly, 2 μg protein of the sample homogenate was loaded onto each well containing Acetyl Co-A reagent. Reactions were initiated by the addition of an oxaloacetate reagent, followed by a measurement of absorbance at 412 nm in a plate reader every 30 s for 20 min at 25 °C. The reaction rate was calculated from the linear portion of the assay curve. The average value of the three biological replicates was calculated.

### 2.6. Immunofluorescence Staining

After 48 h in fixative, the GAS tissues were washed in PBS and stored in 70% ethanol at 4 °C. Tissues were then processed in Leica TP1020 automatic tissue processor (Leica Biosystems, Richmond, IL, USA) and subsequently embedded into paraffin blocks (HistoCore Arcadia C, Leica Biosystems, Richmond, IL, USA). The paraffin-embedded tissue blocks were sliced into 5 μm thick cross-sections using HistoCore Rotary Microtome (Leica Biosystems, Richmond, IL, USA). Tissue sections were deparaffinized and rehydrated in series of Histoclear and ethanol. Antigens were unmasked by heating in Antigen Retrieval Citra, pH-6.0 (#HK086-9K, BioGenex, Fremont, CA, USA) for 20 min using a vegetable steamer. After three washes in PBS, tissue sections were incubated in PowerBlock™ (#HK085-5K, BioGenex) according to the product directions. The sections were then incubated overnight at 4 °C in the following antibodies: myosin, slow and citrate synthase. Details on the primary antibodies and dilution are shown in the Supplementary Table S1. After three washes in PBS, sections were incubated in appropriate secondary antibodies for one hour at room temperature. Alexa Fluor™ 488 goat anti-mouse IgG (#A11001, Invitrogen, Waltham, MA, USA) was used against the myosin and slow antibodies, and Alexa Fluor™ 555 goat anti-rabbit IgG (#A21428, Invitrogen) was used against citrate synthase antibodies. Sections were washed again in PBS three times and mounted with ProLong^™^ Gold Antifade Mountant with DAPI for nuclear staining (#P36931, Invitrogen). All stained sections were viewed with a fluorescence microscope using the 40x objective (Revolve, ECHO, San Diego, CA, USA).

### 2.7. Statistical Analyses

All values are given as the mean ± standard error of the mean (SEM). A two-way analysis of variance (ANOVA) combined with Tukey’s test was used for multiple comparisons among groups (GraphPad Prism, San Diego, CA, USA). A value of *p* < 0.05 was considered to be statistically significant. 

## 3. Results

### 3.1. Western-Style Diet Increases Body Weight Regardless of Moderate-Intensity Exercise Treatment

**Figure 1:**
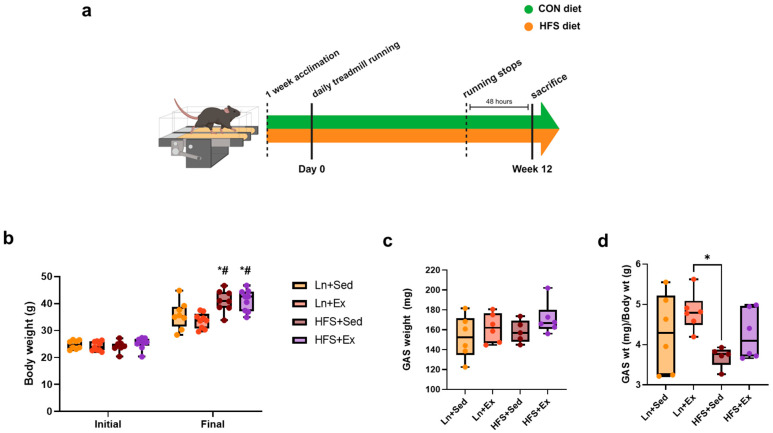
Western-style diet increases body weight regardless of moderate-intensity exercise treatment. (**a**) Schematics of the animal diet and exercise timeline. (**b**) Mean (±SEM) of the initial and final body weight; * *p* < 0.05, initial Ln + Sed vs. final HFS + Sed and HFS + Ex; # *p* < 0.05, initial Ln + Ex vs. final HFS + Sed and HFS + Ex. (**c**) Mean of gastrocnemius (GAS) muscle weight. (**d**) Ratio of GAS weight to total body mass; * *p* < 0.05, Ln + Ex vs. HFS + Sed. Data shown as means ± SEM, *n* = 3 per group.

Figure 1a shows the 12-week timeline of the diet and exercise protocol. Briefly, C57BL/6N mice were either fed a normal rodent chow (CON) diet or a western-style diet composed of high-fat diet with tap water mixed with fructose and sucrose (HFS). After one week of acclimation, daily exercise was implemented until 48 h prior to euthanasia to prevent an acute effect of exercise. At the end of the study period, there was a significant increase in body weight of the mice in the obese group regardless of the exercise protocol (Figure 1b). Although we measured the weight of the GAS muscles and found no significant differences between the groups (Figure 1c), the ratio of this muscle weight to the overall body mass showed a marginal decrease in the obese group, although the difference did not reach a statistical significance relative to the lean control group (Figure 1d).

### 3.2. Exercise Enhances Mitochondrial Function in the Muscle of Obese Animals

**Figure 2:**
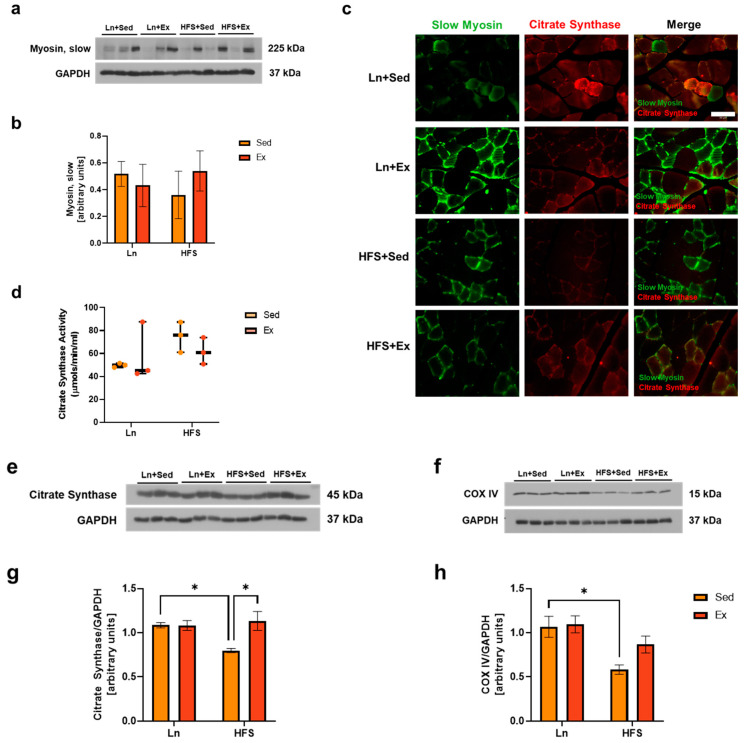
Exercise rescues mitochondrial loss from the GAS muscle of obese animals. (**a**) Representative immunoblots of slow myosin. (**b**) Densitometry analysis of slow myosin levels. (**c**) Co-immunofluorescence staining for slow myosin and citrate synthase on GAS muscle sections, 20×; scale bar 50 μm. (**d**) Mean (± SEM) level of citrate synthase enzyme activity in GAS muscle. (**e**,**f**) Representative immunoblots of citrate synthase and COX IV, respectively. (**g**) Densitometry analysis of citrate synthase; * *p* < 0.05, Ln + Sed vs. HFS + Sed and HFS + Sed vs. HFS + Ex. (**h**) Densitometry analysis of COX IV; * *p* < 0.05, Ln + Sed vs. HFS + Sed. Data shown as means ± SEM, *n* = 3 per group.

Obese individuals exhibited reduced type I fiber and higher glycolytic IIB fiber compared to their lean counterparts [5,12]. In this study, the relative abundance of slow oxidative fiber within GAS muscle tissue did not reach statistical significance between the experimental groups (Figure 2b). To gain further insights into this trend, we directed our attention to the mitochondrial content and its function within the slow fibers. As citrate synthase is a validated biomarker for mitochondrial density in skeletal muscle [9], we aimed to observe citrate synthase in slow fibers. Co-immunofluorescence staining analysis was performed, in which slow-twitch fiber was labeled with an antibody against slow myosin and mitochondria were labeled with an antibody against citrate synthase. The co-localization of the two biomarkers (merge) confirmed the presence of mitochondria within the slow-twitch fibers (Figure 2c). Because a long-term high-fat diet has been shown to increase mitochondrial fatty acid oxidation in muscles [21], citrate synthase enzyme activity was analyzed. In our study, the enzymatic activity of citrate synthase in GAS muscle showed no significant differences across the groups (Figure 2d). In contrast, the protein levels of citrate synthase, as well as that of another mitochondrial content and oxidative metabolism marker, cytochrome *c* oxidase subunit (COX) IV [22], showed a significant reduction in the HFS + Sed group (Figure 2g,h; * *p* < 0.05, Ln + Sed vs. HFS + Sed). The reduced protein expression of citrate synthase was effectively increased in the exercised group (Figure 2g; *p < 0.05, Ln + Sed vs. HFS + Ex). 

### 3.3. Moderate-Intensity Exercise Enhances Mitochondrial Biogenesis Independently from AMPK/PGC-1α Signaling Pathway

**Figure 3:**
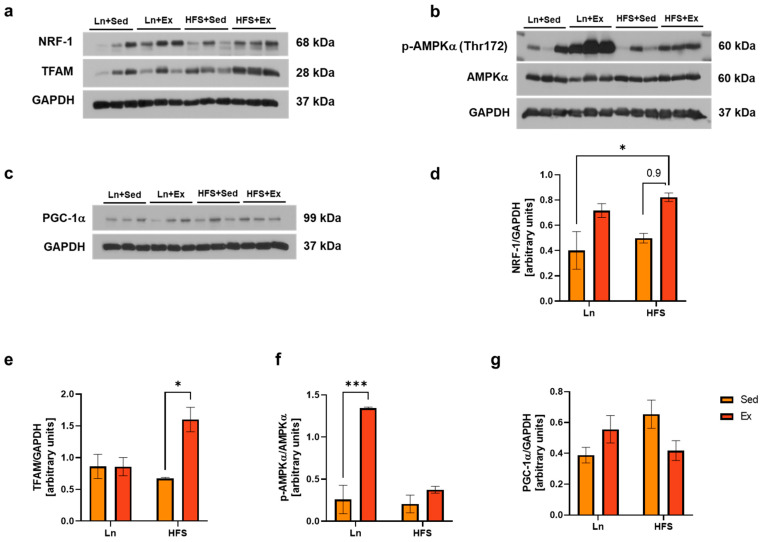
Moderate-Intensity Exercise Enhances Mitochondrial Biogenesis Independently from AMPK/PGC-1α Signaling Pathway. Representative immunoblots of NRF-1 and TFAM (**a**), p-AMPKα (Thr172) and total AMPKα (**b**), and PGC-1α (**c**). (**d**) Densitometry analysis of NRF-1; * *p* < 0.05, Ln + Sed vs. HFS + Ex. (**e**) Densitometry analysis of TFAM; * *p* < 0.05, HFS + Sed vs. HFS + Ex. (**f**) Densitometry analysis of p-AMPK (Thr172) over total AMPK; *** *p* < 0.001, Ln + Sed vs. Ln + Ex. (**g**) Densitometry analysis of PGC-1α; no statistical significance was found. Data shown as means ± SEM, *n* = 3 per group.

Accumulated evidence suggest that exercise triggers the regulation of coactivators and transcription factors such as PGC-1α in the cellular cytosol [22]. The exercise-induced expression of PGC-1α is mediated, in part, by phosphorylation (activation) of AMPK [23]. Following activation, PGC-1α interacts with a variety of transcription factors involved in mitochondrial biogenesis [24]. Hence, we questioned whether the increase in citrate synthase and COX IV in the exercised obese group was associated with mitochondrial biogenesis. As a result, there were increased levels of both mitochondrial biogenesis markers, NRF-1 (Figure 3d; * *p* < 0.05, Ln vs. HFS + Ex) and TFAM (Figure 3e; * *p* < 0.05, HFS vs. HFS + Ex) in the exercised obese group. Intriguingly, the observed increases did not correspond with the expected levels of p-AMPK and PGC-1α, which were hypothesized to be the major signaling molecules involved in the mitochondrial biogenesis pathway. This discrepancy prompted us to speculate about the possibility of alternative pathways that might be responsible for stimulating these biogenesis markers.

### 3.4. DRP-1 Plays a Central Role in Stimulating Both Mitochondrial Biogenesis and Mitophagy

**Figure 4:**
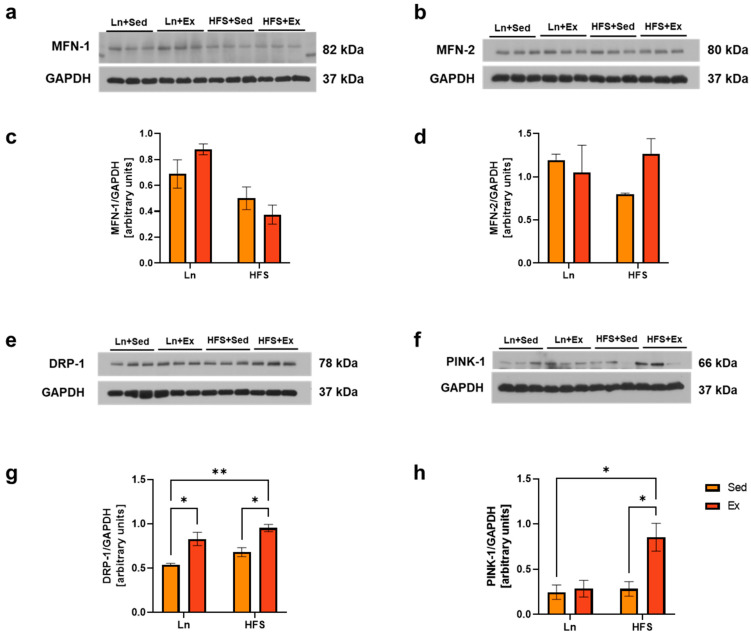
DRP-1 plays a Central Role in Stimulating Both Mitochondrial Biogenesis and Mitophagy. (**a**) Representative immunoblots of MFN-1. (**b**) Representative immunoblots of MFN-2. (**c**) Densitometry analysis of MFN-1; no significance was observed. (**d**) Densitometry analysis of MFN-2; no significance was observed. (**e**,**f**) Representative immunoblots of DRP-1 and PINK-1. (**g**) Densitometry analysis of DRP-1; * *p* < 0.05, Ln + Sed vs. Ln + Ex and HFS + Sed vs. HFS + Ex; ** *p* < 0.01, Ln + Sed vs. HFS + Ex. (**h**) Densitometry of PINK-1; * *p* < 0.05, Ln + Sed vs. HFS + Ex and HFS + Sed vs. HFS + Ex. Data shown as means ± SEM, *n* = 3 per group.

The morphology and structure of mitochondrial organelles are maintained through constant cycles of fusion and fission processes [25]. Mitochondrial fusion, which allows the sharing of healthy mitochondrial content with a damaged organelle, is regulated by dynamin-related GTPases, including mitofusin-1 (MFN-1) and MFN-2 [25,26]. On the other hand, the fission process is mediated by dynamin-related protein-1 (DRP-1), which interacts with several other mitochondrial outer membrane receptors such as the mitochondrial fission factor (MFF) and fission protein-1 (FIS-1) [25,26]. The fission process facilitates the mitophagy of any dysfunctional mitochondria through a process mediated by PTEN-induced kinase-1 (PINK-1) and specific receptors [27]. Therefore, the balance between mitochondrial fusion and fission is crucial for maintaining a healthy pool of mitochondria in skeletal muscle [25]. Obesity has been shown to impair these processes, leading to reduced mitochondrial content and an increased number of dysfunctional mitochondria in skeletal muscle. The results of our study show that obesity did not affect the fusion and fission proteins (Figure 4c,d,g). Interestingly, DRP-1 levels were elevated in both the lean and the obese groups following moderate-intensity exercise (Figure 4g; * *p* < 0.05, Ln + Sed vs. Ln + Ex, HFS + Sed vs. HFS + Ex, and Ln + Sed vs. HFS + Ex). Notably, this increase supports the regulatory role of DRP-1 between mitochondrial biogenesis and mitophagy [26]. The concurrent increase in the mitophagy marker PINK-1 further supports the interrelation of these mitochondrial processes (Figure 4h; * *p* < 0.05, Ln + Sed vs. HFS + Ex and HFS + Sed vs. HFS + Ex).

## 4. Discussion

Obesity has numerous detrimental systemic effects on the body, contributing to the development of the metabolic syndrome, including type 2 diabetes mellitus (T2DM), Alzheimer’s disease, various types of cancer, and cardiovascular diseases. Along with these systemic effects, fat accumulation in vital organs such as the liver (hepatic steatosis), skeletal muscle (myosteatosis), and heart (obesity cardiomyopathy) can adversely impact its respective tissue and lead to poor health outcomes [28]. Notably, an inverse correlation between fatty liver index and muscle density was reported in a population-based prospective cohort study of both male and female subjects [28]. Furthermore, for more than three decades, a substantial number of studies have been reporting an inverse association between the proportion of type I fibers and increased body mass [11]. While it is not clear whether the alteration in fiber type distribution in the muscle of obese individuals is a cause or a consequence of obesity [11], recent studies with human models have reported a reduction of type I fibers in obese individuals. This phenomenon is linked to the metabolic environment associated with obesity, particularly concerning the content and function of mitochondria [10,11].

It has been documented that the accumulation of lipid is greater in type I fibers than type II fibers [11]. This is because the oxidative capacity is greater in type I fiber [11]. Thus, loss of type I fibers can be attributed to the decreased oxidative capacity and lipid metabolism often associated with obese individuals. Although our findings did not support a reduction of type I fibers in our obese mouse models, we observed significantly reduced levels of citrate synthase and COX IV in the muscle tissue. While we examined slow-twitch type I fibers, which are characterized by a higher mitochondrial abundance and oxidative function, it is important to note that muscle fibers are highly adaptable and can change in response to varying functional demands and nutrient availability [2]. Notably, dysfunctional mtDNA can alter the contractile apparatus, highlighting the influence of mitochondrial function in skeletal muscle MyHC fiber type [2]. Despite this connection, variations in mitochondrial energy metabolism and biogenesis markers were not accompanied by the corresponding MyHC-type switch, a discovery which is in agreement with findings from previous research [2]. This inconsistency might help elucidate why some obese individuals do not exhibit metabolic abnormalities, such a condition being known as metabolically healthy obesity (MHO). Thus, we propose that future studies examine the muscle fiber composition of obese individuals with or without metabolic complications. Such research could clarify the extent to which muscle fiber type proportions contribute to metabolic health in obesity-affected individuals.

Several human model studies have reported an inverse relationship between type I fiber proportion and body weight, proposing type I fiber as a predictor of obesity [5,12]. Intriguingly, Tanner et al. also reported that morbidly obese individuals with a greater type I fiber proportion tended to lose more body mass following weight loss intervention [12]. In another study with a human model, the muscle mass of endurance-trained individuals had a greater proportion of type I muscle fibers [29]. However, our findings did not show significant difference in the proportion of type I fibers in the sedentary or exercised obese groups. A possible explanation may be that endurance exercise training has the ability to increase the oxidative potential of both glycolytic and oxidative fibers [30].

Reduction of the muscle ATP/ADP ratio following exercise can activate AMPK levels, integrating physiological exercise signals with the regulation of the muscle mitochondrial system [31,32]. AMPK-induced muscle mitochondrial function is mediated, in part, by PGC-1α signaling, which, subsequently, promotes mitochondrial biogenesis and the quality control system through coactivating a number of nuclear receptors [31]. In our study, exercise only increased the activation of AMPK in the muscles of the lean control group, but not in the obese group. We speculate that this phenomenon might be due to the elevated ATP/ADP ratio associated with an obese state, something which is also implied by previous studies [33]. Furthermore, the consistent levels of PGC-1α in our findings suggest that there may be other factors associated with exercise-induced mitochondrial biogenesis. Concurrently with our findings, it has been reported that PGC-1α is dispensable for endurance-exercise-induced mitochondrial biogenesis in skeletal muscle [24]. Thus, we cannot rule out mitochondrial adaptation to other forms of exercise, such as resistance training, which might be regulated by PGC-1α [13,24].

TFAM is an important transcription factor involved in mitochondrial biogenesis as it regulates mtDNA replication, repair, and gene transcription [34]. TFAM has also been suggested to attenuate high-fat-diet-induced fat accumulation and insulin resistance in skeletal muscle [34]. The upregulation of TFAM, NRF-1, and DRP-1 was not a surprising finding, as previous studies have also shown upregulation of mitochondrial biogenesis and fission markers in mice after exercise training [35]. The fission of mitochondria results in the creation of smaller organelles, allowing the one with high membrane potential to undergo efficient engulfment by autophagosome [13,36]. This fission and removal process play an important role in mitochondrial homeostasis promoting mitochondrial turnover [13]. It is noteworthy that type I and IIA fibers exhibit an elongated mitochondrial reticulum and higher rates of mitochondrial fusion compared to type IIB/X fibers [37]. The observed discrepancy in our study between mitochondrial oxidative markers and dynamic proteins underscores the necessity of conducting individual analyses of these fibers in the context of the exercise protocol. This approach will provide a more comprehensive understanding of the alterations to dynamic markers within specific muscle fiber types.

**Figure 5:**
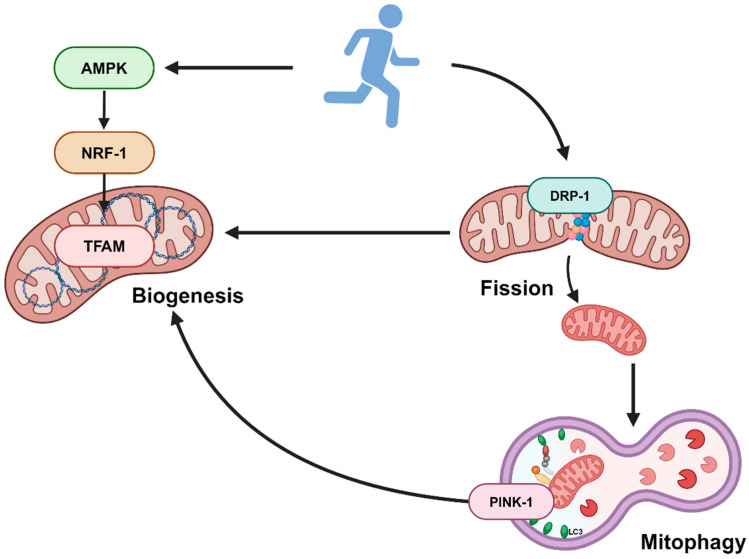
Schematics of study findings. Created with Biorender.com.

Physical activity increases mitochondrial turnover by activating the fission and mitophagy process, which is associated with better mitochondrial quality and efficient removal of dysfunctional mitochondria in skeletal muscle [36,38]. Notably, it has been shown that, after 90 min of acute exercise, changes in the morphology of mitochondria were visible in electron micrograph images, with smaller and more round structures along specific areas of the muscle z-line [35]. This indicates that the increased DRP-1 levels in our study may have resulted from the intermyofibrillar mitochondria. Given that it plays a central role between mitochondrial biogenesis and dynamics, DRP-1 could be explored as a therapeutic target for mitigating the mitochondrial dynamics and reduced oxidative capacity associated with obesity. The findings from our current study is summarized in Figure 5.

Numerous studies have investigated potential pharmacological interventions that mimic the effects of exercise. These mimetics, including resveratrol, rapamycin, and metformin [39], have been shown to target muscle mitochondrial biogenesis signaling. However, the ability of these exercise mimetics to increase the quality of skeletal muscle remains questionable, as these mimetics have been shown to inhibit muscle protein synthesis [39]. Furthermore, increases in mitochondrial growth and number cannot alter energy expenditure and must be combined with exercise in order to burn calories [39]. Other beneficial effects of exercise such as improving cognitive and bone health and reducing the risk of developing several chronic diseases should be considered as well [40]. Taken together, lifestyle interventions such as moderate-intensity exercise and a healthy diet remain the initial and foremost treatment strategy to effectively combat the obesity-related health outcomes.

There are several limitations to this study worth noting. Our study included a sample size of *n* = 3; however, this did not impact the statistical power. With this sample size, we nonetheless show robust differences in the levels of target proteins between groups. Our study observed great variability in the relative abundance of slow-twitch fibers within each group. This may be due to several factors such as biological variability and sample preparation. Moreover, as with other research methodologies, the analysis of type I fiber focuses on “pure” muscle fibers characterized by one isoform of MyHC [11]. It needs to be considered that skeletal muscle contains a variable portion of “hybrid” fibers that exhibit a mixture of MyHC isoforms [11]. Thus, we recommend that future studies implement additional experimental methods to examine metabolic changes as well as changes in the MyHC isoform within specific muscle fiber types using a single-fiber analysis. Additionally, proteomics analyses may provide a deeper understanding of the biological mechanisms responsible for metabolic alterations in obese models. In this current study, GAS muscle was not divided into red and white subtypes. However, because these subtypes of muscle fibers exhibit distinct metabolic and contractile properties, analyzing the red and white portions separately could potentially lead to differential responses to obesity and exercise stimuli. Another limitation in our study consists of the inability to visualize mitochondrial morphology and structure using transmission electron microscopy (TEM), which allows discrete measurements of mitochondrial morphology and structure in the subsarcolemmal and intermyofibrillar compartments. 

## 5. Conclusions

In summary, obesity has been shown to reduce type I fiber proportions in skeletal muscle; however, whether the loss of this fiber is a cause or a consequence of obesity remains to be elucidated. In our study, we report that loss of mitochondrial content occurs prior to the loss of type I fibers in obese murine models. Additionally, we report that moderate-intensity exercise can prevent the loss of mitochondrial content by promoting mitochondrial biogenesis. Interestingly, the exercise-induced mitochondrial biogenesis was shown to be independent of the AMPK/PGC-1α signaling pathway. We propose that moderate-intensity exercise promotes the production of the mitochondrial fission protein, DRP-1, which can subsequently activate mitochondrial biogenesis as well as mitophagy. This, in turn, enhances the mitochondrial quality control system and increases the number and oxidative metabolism of mitochondria in obese models. Restoring the proportion of type I fibers in muscle tissue is crucial to mitigate muscle and whole-body pathophysiology, thereby improving impaired metabolism and function in obese individuals. Therefore, our study provides insights into the mechanisms by which obesity impairs muscle metabolism and into how exercise counteracts these deleterious effects.

## Data Availability

Data are contained within the article.

## Supplementary Materials

The following supporting information can be downloaded at: https://www.mdpi.com/article/10.3390/nu16121836/s1, Table S1: List of antibodies.
