# Supplementary material for: Moderate-Intensity Exercise Enhances Mitochondrial Biogenesis Markers in the Skeletal Muscle of a Mouse Model Affected by Diet-Induced Obesity
Source: Nutrients. 2024 Jun 12;16(12):1836. doi: 10.3390/nu16121836 (PMC11206830; doi:10.3390/nu16121836)
Supplement: Supplementary file 1 [file nutrients-16-01836-s001.zip › nutrients-2992569-supplementary.pdf]

Supplemental Table S1. List of antibodies used for Western Blot and Immunofluorescence Staining. The manufacturer, identifier number, host species, and dilution are indicated.

Antibodies (WB)	Source	Identifier	Host	Dilution
Myosin, slow	Sigma	M8421	Mouse	1:5000
COX IV	Cell Signaling	4850	Rabbit	1:1000
Citrate Synthase	Invitrogen	PA5-22126	Rabbit	1:1000
NRF-1	Cell Signaling	69432	Rabbit	1:1000
TFAM	Santa Cruz	SC-166965	Mouse	1:1000
p-AMPK	Cell Signaling	2535	Rabbit	1:1000
AMPK	Cell Signaling	2532	Rabbit	1:1000
PGC-1α	Invitrogen	PA572948	Rabbit	1:1000
DRP-1	Cell Signaling	5391	Rabbit	1:1000
MFN-2	Cell Signaling	9482	Rabbit	1:1000
PINK1	Santa Cruz	SC-517353	Mouse	1:1000
GAPDH	Invitrogen	MA5-15738	Mouse	1:2000
Anti-Mouse IgG	Enzo Life Sciences	ADI-SAB-100-J	Goat	
Anti-Rabbit IgG	Enzo Life Sciences	ADI-SAB-300-J	Goat	
**Antibodies (WB)**	**Source**	**Identifier**	**Host**	**Dilution**
Myosin, slow	Sigma	M8421	Mouse	1:1000
Citrate Synthase	Invitrogen	PA5-22126	Rabbit	1:100

Abbreviations

COX IV, Cytochrome *c* oxidase subunit 4; NRF, Nuclear factor erythroid 2-related factor; TFAM, Transcription factor A, Mitochondrial; AMPK, AMP-activated protein kinase; PGC-1α, Peroxisome proliferator-activated receptor gamma coactivator 1-alpha; DRP, Dynamin-related protein; MFN, Mitofusin; PINK, PTEN induced putative kinase; GAPDH, Glyceraldehyde 3-phosphate dehydrogenase.
